# Supplementary material for: Diagnosis of Progressive Disseminated Histoplasmosis in Advanced HIV: A Meta-Analysis of Assay Analytical Performance
Source: J Fungi (Basel). 2019 Aug 18;5(3):76. doi: 10.3390/jof5030076 (PMC6787751; doi:10.3390/jof5030076)
Supplement: Supplementary file 1 [file jof-05-00076-s001.zip › proof back suppl/jof-576549-supplemntary material 1.pdf]

Search for histoplasmosis, HIV, and antigen testing

Database	Strategy	Run Date	Records
Medline (Ovid) 1946-	(histoplasmos* OR histoplasma).mp. OR exp Histoplasmosis/<br><br>AND<br><br>(hiv OR human immunodeficiency virus OR aids OR acquired immunodeficiency syndrome OR immunodeficiency).mp. OR exp HIV/ OR exp Acquired Immunodeficiency Syndrome/<br><br>AND<br><br>(antigen* OR immune complex* OR autoantigen* OR epitope* OR isoantigen* OR superantigen* OR allergen* OR histoplasmin OR serolog* OR serum* OR blood OR plasma OR marrow* OR mycolog* OR PCR OR polymerase chain reaction*).mp. OR exp Antigens/ OR exp Blood/ OR exp Bone Marrow/ OR exp Serology/ OR exp Mycology/ OR exp Polymerase Chain Reaction/<br><br>AND<br><br>(sensitiv* OR diagnos* OR predictive value*).mp. OR accurac*.ti,ab.<br><br>Limits: English, Spanish, Portuguese	2/20/19	366 articles
Embase (Ovid) 1947-	(histoplasmos* OR histoplasma).mp. OR exp histoplasmosis/<br><br>AND<br><br>(hiv OR human immunodeficiency virus OR aids OR acquired immunodeficiency syndrome OR immunodeficiency).mp. OR exp Human immunodeficiency virus/ OR exp acquired immune deficiency syndrome/<br><br>AND<br><br>(antigen* OR immune complex* OR autoantigen* OR epitope* OR isoantigen* OR superantigen* OR allergen* OR histoplasmin OR serolog* OR serum* OR blood OR plasma OR marrow* OR mycolog* OR PCR OR polymerase chain reaction*).mp. OR exp antigen/ OR exp blood/ OR exp bone marrow/ OR exp serology/ OR exp mycology/ OR exp polymerase chain reaction/<br><br>AND	2/20/19	1028 articles<br><br>-303 duplicates<br><br>=725 articles

	(sensitiv* OR diagnos* OR predictive value*).mp. OR accurac*.ti,ab.<br><br>Limits: English, Spanish, Portuguese		
CAB Abstracts (Ovid)<br>1910-	(histoplasmos* OR histoplasma).mp. OR exp histoplasmosis/<br><br>AND<br><br>(hiv OR human immunodeficiency virus OR aids OR acquired immunodeficiency syndrome OR immunodeficiency).mp. OR exp human immunodeficiency viruses/ OR exp acquired immune deficiency syndrome/<br><br>AND<br><br>(antigen* OR immune complex* OR autoantigen* OR epitope* OR isoantigen* OR superantigen* OR allergen* OR histoplasmin OR serolog* OR serum* OR blood OR plasma OR marrow* OR mycolog* OR PCR OR polymerase chain reaction*).mp. OR exp antigens/ OR exp blood/ OR exp bone marrow/ OR exp serology/ OR exp mycology/ OR exp polymerase chain reaction/<br><br>AND<br><br>(sensitiv* OR diagnos* OR predictive value*).mp. OR accurac*.ti,ab.<br><br>Limits: English, Spanish, Portuguese	2/20/19	270 articles<br><br>-197 duplicates<br><br>=73 articles
Global Health (Ovid)<br>1910-	(histoplasmos* OR histoplasma).mp. OR exp histoplasmosis/<br><br>AND<br><br>(hiv OR human immunodeficiency virus OR aids OR acquired immunodeficiency syndrome OR immunodeficiency).mp. OR exp human immunodeficiency viruses/ OR exp acquired immune deficiency syndrome/<br><br>AND<br><br>(antigen* OR immune complex* OR autoantigen* OR epitope* OR isoantigen* OR superantigen* OR allergen* OR histoplasmin OR serolog* OR serum* OR blood OR plasma OR marrow* OR mycolog* OR PCR OR polymerase chain reaction*).mp. OR exp antigens/ OR exp blood/ OR exp bone marrow/ OR exp serology/ OR exp mycology/ OR exp polymerase chain reaction/	2/20/19	273 articles<br><br>-195 duplicates<br><br>=2 articles

	AND<br><br>(sensitiv* OR diagnos* OR predictive value*).mp. OR accurac*.ti,ab.<br><br>Limits: English, Spanish, Portuguese		
Scopus<br>1960-	TITLE-ABS-KEY (histoplasmos* OR histoplasma) AND TITLE-ABS-KEY (hiv OR "human immunodeficiency virus" OR aids OR "acquired immunodeficiency syndrome" OR immunodeficiency) AND TITLE-ABS-KEY(antigen* OR "immune complex" OR autoantigen* OR epitope* OR isoantigen* OR superantigen* OR allergen* OR histoplasmin OR serolog* OR serum* OR blood OR plasma OR marrow* OR mycolog* OR PCR OR "polymerase chain reaction") AND (TITLE-ABS-KEY (sensitiv* OR diagnos* OR "predictive value") OR TITLE-ABS (accurac*)) AND (LIMIT-TO (LANGUAGE , "English") OR LIMIT-TO (LANGUAGE , "Spanish") OR LIMIT-TO (LANGUAGE , "Portuguese"))	2/20/19	584 articles<br><br>-554 duplicates<br><br>=31 articles
Cochrane Library<br>1800-	#1(histoplasmos* OR histoplasma):ti,ab,kwLimits 93<br>#2MeSH descriptor: [Histoplasmosis] explode all treesMeSH 42<br>#3MeSH descriptor: [HIV] explode all treesMeSH 2896<br>#4MeSH descriptor: [Acquired Immunodeficiency Syndrome] explode all treesMeSH 1709<br>#5(hiv OR "human immunodeficiency virus" OR aids OR "acquired immunodeficiency syndrome" OR immunodeficiency):ti,ab,kwLimits 23558<br>#6(sensitiv* OR diagnos* OR "predictive value"):ti,ab,kwLimits 201636<br>#7(accurac*):ti,abLimits 13660<br>#8(antigen* OR "immune complex" OR autoantigen* OR epitope* OR isoantigen* OR superantigen* OR allergen* OR histoplasmin OR serolog* OR serum* OR blood OR plasma OR marrow* OR mycolog* OR PCR OR "polymerase chain reaction"):ti,ab,kwLimits 342426<br>#9MeSH descriptor: [Antigens] explode all treesMeSH 13620<br>#10MeSH descriptor: [Blood] explode all treesMeSH 15019<br>#11MeSH descriptor: [Bone Marrow] explode all treesMeSH 595<br>#12MeSH descriptor: [Serology] explode all treesMeSH 5<br>#13MeSH descriptor: [Mycology] explode all treesMeSH 10<br>#14MeSH descriptor: [Polymerase Chain Reaction] explode all treesMeSH 2171	2/20/19	7 articles<br><br>-6 duplicates<br><br>=1 article

	#15#1 OR #2Limits 93<br>#16#3 OR #4 OR #5Limits 23558<br>#17#6 OR #7Limits 207398<br>#18#8 OR #9 OR #10 OR #11 OR #12 OR #13 OR #14Limits 345397<br>#19#15 AND #16 AND #17 AND #18Limits 7<br><br>Limits: English, Spanish, Portuguese		
PubMed Central (PMC) 2000-	((((((((histoplasmos*[Abstract] OR histoplasma[Abstract])) OR (histoplasmos*[Title] OR histoplasma[Title])) OR "histoplasmosis"[MeSH Terms])) AND ((((hiv [Abstract] OR "human immunodeficiency virus" [Abstract] OR aids [Abstract] OR "acquired immunodeficiency syndrome" [Abstract] OR immunodeficiency[Abstract])) OR (hiv [Title] OR "human immunodeficiency virus" [Title] OR aids [Title] OR "acquired immunodeficiency syndrome" [Title] OR immunodeficiency[Title])) OR ("hiv"[MeSH Terms] OR "acquired immunodeficiency syndrome"[MeSH Terms]))) AND (((sensitiv*[Abstract] OR diagnos*[Abstract] OR "predictive value"[Abstract] OR accurac*[Abstract])) OR (sensitiv*[Title] OR diagnos*[Title] OR "predictive value"[Title] OR accurac*[Title]))) AND ((((antigen*[Abstract] OR "immune complex"[Abstract] OR autoantigen*[Abstract] OR epitope*[Abstract] OR isoantigen*[Abstract] OR superantigen*[Abstract] OR allergen*[Abstract] OR histoplasmin[Abstract] OR serolog*[Abstract] OR serum*[Abstract] OR blood[Abstract] OR plasma[Abstract] OR marrow*[Abstract] OR mycolog*[Abstract] OR PCR[Abstract] OR "polymerase chain reaction"[Abstract])) OR (antigen*[Title] OR "immune complex"[Title] OR autoantigen*[Title] OR epitope*[Title] OR isoantigen*[Title] OR superantigen*[Title] OR allergen*[Title] OR histoplasmin[Title] OR serolog*[Title] OR serum*[Title] OR blood[Title] OR plasma[Title] OR marrow*[Title] OR mycolog*[Title] OR PCR[Title] OR "polymerase chain reaction"[Title])) OR ("Antigens"[Mesh] OR "Blood"[Mesh] OR "Bone Marrow"[Mesh] OR "Mycology"[Mesh] OR "Serology"[Mesh] OR "Polymerase Chain Reaction"[Mesh])))) NOT ((((((((histoplasmos*[Abstract] OR histoplasma[Abstract])) OR (histoplasmos*[Title] OR histoplasma[Title])) OR "histoplasmosis"[MeSH Terms])) AND ((((hiv [Abstract] OR "human immunodeficiency virus" [Abstract] OR aids [Abstract] OR "acquired immunodeficiency syndrome" [Abstract] OR immunodeficiency[Abstract])) OR (hiv [Title] OR	2/20/19	12 articles<br><br>-10 duplicates<br><br>=2 articles

	"human immunodeficiency virus" [Title] OR aids [Title] OR "acquired immunodeficiency syndrome" [Title] OR immunodeficiency[Title])) OR ("hiv"[MeSH Terms] OR "acquired immunodeficiency syndrome"[MeSH Terms]))) AND (((sensitiv*[Abstract] OR diagnos*[Abstract] OR "predictive value"[Abstract] OR accurac*[Abstract])) OR (sensitiv*[Title] OR diagnos*[Title] OR "predictive value"[Title] OR accurac*[Title]))) AND ((((antigen*[Abstract] OR "immune complex"[Abstract] OR autoantigen*[Abstract] OR epitope*[Abstract] OR isoantigen*[Abstract] OR superantigen*[Abstract] OR allergen*[Abstract] OR histoplasmin[Abstract] OR serolog*[Abstract] OR serum*[Abstract] OR blood[Abstract] OR plasma[Abstract] OR marrow*[Abstract] OR mycolog*[Abstract] OR PCR[Abstract] OR "polymerase chain reaction"[Abstract])) OR (antigen*[Title] OR "immune complex"[Title] OR autoantigen*[Title] OR epitope*[Title] OR isoantigen*[Title] OR superantigen*[Title] OR allergen*[Title] OR histoplasmin[Title] OR serolog*[Title] OR serum*[Title] OR blood[Title] OR plasma[Title] OR marrow*[Title] OR mycolog*[Title] OR PCR[Title] OR "polymerase chain reaction"[Title])) OR ("Antigens"[Mesh] OR "Blood"[Mesh] OR "Bone Marrow"[Mesh] OR "Mycology"[Mesh] OR "Serology"[Mesh] OR "Polymerase Chain Reaction"[Mesh]))) AND medline[sb])		
LILACS	(tw:(histoplasmos* OR histoplasma)) AND (tw:(hiv OR "human immunodeficiency virus" OR aids OR "acquired immunodeficiency syndrome" OR immunodeficiency)) AND (tw:(sensitiv* OR diagnos* OR "predictive value" OR accurac*)) AND (tw:(antigen* OR "immune complex" OR autoantigen* OR epitope* OR isoantigen* OR superantigen* OR allergen* OR histoplasmin OR serolog* OR serum* OR blood OR plasma OR marrow* OR mycolog* OR PCR OR "polymerase chain reaction"))<br><br>Limits: LILACS, English, Spanish, Portuguese	2/20/19	70 articles<br><br>-11 duplicates<br><br>=59 articles
		**De-duplicated in EndNote:**	**1259 articles**

Broad search on histoplasmosis, HIV, and diagnosis

*Diagnostic methodology search filter adapted from:

Haynes RB,Wilczynski NL. Optimal search strategies for retrieving scientifically strong studies of diagnosis from MEDLINE: analytical survey. BMJ. 2004; 328:1040-2.

**Database**	**Strategy**	**Run Date**	**Records**
Medline (Ovid) 1946-	(histoplasmos* OR histoplasma).mp. OR exp Histoplasmosis/<br><br>AND<br><br>(hiv OR human immunodeficiency virus OR aids OR acquired immunodeficiency syndrome OR immunodeficiency).mp. OR exp HIV/ OR exp Acquired Immunodeficiency Syndrome/<br><br>AND<br><br>(sensitiv* OR diagnos* OR predictive value*).mp. OR accurac*.ti,ab.<br><br>Limits: English, Spanish, Portuguese	2/20/19	870 articles
Embase (Ovid) 1947-	(histoplasmos* OR histoplasma).mp. OR exp histoplasmosis/<br><br>AND<br><br>(hiv OR human immunodeficiency virus OR aids OR acquired immunodeficiency syndrome OR immunodeficiency).mp. OR exp Human immunodeficiency virus/ OR exp acquired immune deficiency syndrome/<br><br>AND<br><br>(sensitiv* OR diagnos* OR predictive value*).mp. OR accurac*.ti,ab.<br><br>Limits: English, Spanish, Portuguese	2/20/19	1701 articles<br><br>-724 duplicates<br><br>=977 articles
CAB Abstracts (Ovid) 1910-	(histoplasmos* OR histoplasma).mp. OR exp histoplasmosis/<br><br>AND<br><br>(hiv OR human immunodeficiency virus OR aids OR acquired immunodeficiency syndrome OR immunodeficiency).mp. OR exp human immunodeficiency viruses/ OR exp acquired immune deficiency syndrome/<br><br>AND	2/20/19	507 articles<br><br>- 375 duplicates<br><br>= 132 articles

	(sensitiv* OR diagnos* OR predictive value*).mp. OR accurac*.ti,ab.<br><br>Limits: English, Spanish, Portuguese		
Global Health (Ovid)<br>1910-	(histoplasmos* OR histoplasma).mp. OR exp histoplasmosis/<br><br>AND<br><br>(hiv OR human immunodeficiency virus OR aids OR acquired immunodeficiency syndrome OR immunodeficiency).mp. OR exp human immunodeficiency viruses/ OR exp acquired immune deficiency syndrome/<br><br>AND<br><br>(sensitiv* OR diagnos* OR predictive value*).mp. OR accurac*.ti,ab.<br><br>Limits: English, Spanish, Portuguese	2/20/19	522 articles<br><br>- 514 duplicates<br><br>= 8 articles
Scopus<br>1960-	TITLE-ABS-KEY (histoplasmos* OR histoplasma) AND TITLE-ABS-KEY (hiv OR "human immunodeficiency virus" OR aids OR "acquired immunodeficiency syndrome" OR immunodeficiency) AND (TITLE-ABS-KEY (sensitiv* OR diagnos* OR "predictive value") OR TITLE-ABS (accurac*)) AND (LIMIT-TO (LANGUAGE , "English") OR LIMIT-TO (LANGUAGE , "Spanish") OR LIMIT-TO (LANGUAGE , "Portuguese"))	2/20/19	1119 articles<br><br>- 1058 duplicates<br><br>= 61 articles
Cochrane Library<br>1800-	#1(histoplasmos* OR histoplasma):ti,ab,kw Limits 93<br>#2MeSH descriptor: [Histoplasmosis] explode all treesMeSH 42<br>#3MeSH descriptor: [HIV] explode all treesMeSH 2896<br>#4MeSH descriptor: [Acquired Immunodeficiency Syndrome] explode all treesMeSH 1709<br>#5(hiv OR "human immunodeficiency virus" OR aids OR "acquired immunodeficiency syndrome" OR immunodeficiency):ti,ab,kw Limits 23558<br>#6(sensitiv* OR diagnos* OR "predictive value"):ti,ab,kw Limits 201636<br>#7(accurac*):ti,abLimits 13660<br>#8#1 OR #2Limits 93<br>#9#5 OR #3 OR #4Limits 23558<br>#10#6 OR #7Limits 207398<br>#11#8 AND #9 AND #10Limits 7<br><br>Limits: English, Spanish, Portuguese	2/20/19	7 articles<br><br>- 6 duplicates<br><br>= 1 article
PubMed Central (PMC)	((((((((histoplasmos*[Title] OR histoplasma[Title])) OR (histoplasmos*[Abstract] OR	2/20/19	49 articles

2000-	histoplasma[Abstract])) OR "histoplasmosis"[MeSH Terms])) AND ((((hiv [Title] OR "human immunodeficiency virus" [Title] OR aids [Title] OR "acquired immunodeficiency syndrome" [Title] OR immunodeficiency[Title])) OR (hiv [Abstract] OR "human immunodeficiency virus" [Abstract] OR aids [Abstract] OR "acquired immunodeficiency syndrome" [Abstract] OR immunodeficiency[Abstract])) OR ("hiv"[MeSH Terms] OR "acquired immunodeficiency syndrome"[MeSH Terms]))) AND (((sensitiv*[Title] OR diagnos*[Title] OR "predictive value"[Title] OR accurac*[Title])) OR (sensitiv*[Abstract] OR diagnos*[Abstract] OR "predictive value"[Abstract] OR accurac*[Abstract])))) NOT ((((((((histoplasmos*[Title] OR histoplasma[Title])) OR (histoplasmos*[Abstract] OR histoplasma[Abstract])) OR "histoplasmosis"[MeSH Terms])) AND ((((hiv [Title] OR "human immunodeficiency virus" [Title] OR aids [Title] OR "acquired immunodeficiency syndrome" [Title] OR immunodeficiency[Title])) OR (hiv [Abstract] OR "human immunodeficiency virus" [Abstract] OR aids [Abstract] OR "acquired immunodeficiency syndrome" [Abstract] OR immunodeficiency[Abstract])) OR ("hiv"[MeSH Terms] OR "acquired immunodeficiency syndrome"[MeSH Terms]))) AND (((sensitiv*[Title] OR diagnos*[Title] OR "predictive value"[Title] OR accurac*[Title])) OR (sensitiv*[Abstract] OR diagnos*[Abstract] OR "predictive value"[Abstract] OR accurac*[Abstract]))) AND medline[sb])		- 45 duplicates<br><br>= 4 articles
LILACS	(tw:(histoplasmos* OR histoplasma)) AND (tw:(hiv OR "human immunodeficiency virus" OR aids OR "acquired immunodeficiency syndrome" OR immunodeficiency)) AND (tw:(sensitiv* OR diagnos* OR "predictive value" OR accurac*)) AND (instance:"regional")<br><br>Limits: LILACS, English, Spanish, Portuguese	2/20/19	210 articles<br><br>- 35 duplicates<br><br>= 175 articles
		**De-duplicated in EndNote:**	**2228 articles**
